# Diet-Induced Gut Dysbiosis and Leaky Gut Syndrome

**DOI:** 10.4014/jmb.2312.12031

**Published:** 2024-02-01

**Authors:** Yu-Rim Chae, Yu Ra Lee, Young-Soo Kim, Ho-Young Park

**Affiliations:** 1 Food Functionality Research Division, Korea Food Research Institute, Jeollabuk-do 55365, Republic of Korea; 2 Department of Food Science and Technology, Jeonbuk National University, Jeollabuk-do 54896, Republic of Korea; 3 Department of Food Biotechnology, Korea National University of Science and Technology, Daejeon 34113, Republic of Korea

**Keywords:** Diet, leaky gut syndrome, microbiota, intestinal dysbiosis, tight junction proteins

## Abstract

Chronic gut inflammation promotes the development of metabolic diseases such as obesity. There is growing evidence which suggests that dysbiosis in gut microbiota and metabolites disrupt the integrity of the intestinal barrier and significantly impact the level of inflammation in various tissues, including the liver and adipose tissues. Moreover, dietary sources are connected to the development of leaky gut syndrome through their interaction with the gut microbiota. This review examines the effects of these factors on intestinal microorganisms and the communication pathways between the gut-liver and gut-brain axis. The consumption of diets rich in fats and carbohydrates has been found to weaken the adherence of tight junction proteins in the gastrointestinal tract. Consequently, this allows endotoxins, such as lipopolysaccharides produced by detrimental bacteria, to permeate through portal veins, leading to metabolic endotoxemia and alterations in the gut microbiome composition with reduced production of metabolites, such as short-chain fatty acids. However, the precise correlation between gut microbiota and alternative sweeteners remains uncertain, necessitating further investigation. This study highlights the significance of exploring the impact of diet on gut microbiota and the underlying mechanisms in the gut-liver and gut-brain axis. Nevertheless, limited research on the gut-liver axis poses challenges in comprehending the intricate connections between diet and the gut-brain axis. This underscores the need for comprehensive studies to elucidate the intricate gut-brain mechanisms underlying intestinal health and microbiota.

## Introduction

The microbiota is a diverse and intricate ecosystem primarily composed of bacteria but also encompassing viruses, fungi, protozoa, and archaea [1]. These microorganisms play a crucial role in multiple facets of human physiology, encompassing dietary habits, metabolic functions, defense against pathogens, safeguarding the intestinal barrier, maturation of the immune system, and the preservation of immune equilibrium [2]. The gut microbiota has a significant impact on the overall health of its host, and an imbalance in the gut microbiota, known as gut dysbiosis, is now widely acknowledged as a prominent characteristic of obesity and various metabolic disorders [3]. This review provides a concise overview of research findings that suggest a possible connection between diet and the development of leaky gut syndrome, obesity, and metabolic disorders through the modulation of the gut microbiota.

## Relationship between Diet and Microbiota

In human gut microflora, there are around 10^13^ different types of microorganisms in the intestinal mucosa. *Firmicutes* and *Bacteroidetes* constitute approximately 90% of the gut microbiome, with *Actinobacteria*, *Proteobacteria*, *Fusobacteria*, and *Verrucomicrobia* constituting the other major phyla of gut bacteria [4]. Of these, there are 500–1000 different species (spp.). Bacteria are prevalent, and their combined genomes are expected to include 100 times as many genes as the mammalian genotype [5].

The microbiota has numerous effects on an individual's well-being, including the activation of the immune system, the breakdown of dietary fibers, increased function and motility of the gastrointestinal tract, and the facilitation of nutrient absorption, as well as protection against infections [6, 7]. Numerous human metabolic processes and clinical parameters could be influenced by gut microbes [8]. The human diet and gut microbiota are closely correlated, and with societal development, there has been an increase in the consumption of refined carbohydrates, Western-style high-fat diets (HFDs), and sugar. Notably, recent research suggests that the diet-microbiota interplay is becoming increasingly personalized, highlighting the need for tailored adjustments based on individual circumstances [9].

## HFD

HFDs are known to not only induce obesity but also cause gut dysbiosis [10]. An imbalance in the gut microbiota can inhibit the expression of proteins responsible for maintaining the integrity of intestinal tight junctions (TJs). By upregulating the expression of TJ proteins, probiotic strains such as *Lactobacillus* and *Bifidobacterium* can potentially mitigate the progression of autoimmune disorders in individuals with genetic predispositions [11]. Consequently, this disruption can result in an increased translocation of lipopolysaccharides (LPSs) from the gut into the bloodstream, resulting in a condition commonly referred to as metabolic endotoxemia [12]. Changes in the composition of gut microbiota populations stimulate the Toll-like receptor signaling pathway, resulting in heightened permeability of the intestines to endotoxins, specifically LPSs. Consequently, this process facilitates the movement of LPSs from the intestines into the bloodstream [13]. These occurrences result in a disparity between the host and microbial communities, compromise barrier function, and are characterized by abnormally high intestinal permeability and altered epithelial TJ molecule expression [14].

Furthermore, HFD induced bile acid production compromises the integrity of the intestinal mucosal barrier. Bile acids facilitate the emulsification of luminal fat, expanding the overall surface area for lipase-driven digestion of micelles, enabling absorption by intestinal epithelial cells [15]. HFD elevates intestinal permeability through direct stimulation of proinflammatory signaling cascades and indirectly by upregulating barrier-disrupting cytokines such as TNFα, interleukin (IL)-1β [16], IL-6 [17], and interferon γ [18], while concurrently reducing the levels of barrier-forming cytokines (IL-10 [19], IL-17 [20] and IL-22 [21]). Ultimately, an HFD unfavorably alters the composition of intestinal mucus and promotes the colonization of the gut microbiota by species known to disrupt the intestinal barrier. In addition to various TJs that impede the permeation of luminal contents, the intestinal barrier incorporates a superficial unstirred mucus layer (SUML). This layer envelops intestinal epithelial cells with a protective environment containing bicarbonate, antimicrobials, IgA, glycoproteins, and lubricant. Notably, studies revealed the indispensable nature of the SUML in gut health, as mice deficient in mucin 2 (MUC2), a glycoprotein constituting the majority of the mucus, exhibited spontaneous colitis, resembling the presentation seen in dextran sulfate-sodium–induced colitis [22].

Junctional adhesion molecules (JAMs) belong to a subset within the immunoglobulin superfamily of adhesion receptors, playing diverse physiological roles in the development and maintenance of homeostasis in vertebrates [23]. While JAM-A is not restricted solely to tight junctions, it is also present along the lateral membrane domain, yet it exhibits a significant enrichment at the tight junctions [24], and JAM-A serves to regulate the barrier function of the epithelium. Several research studies have demonstrated that HFDs elevate the levels of endotoxin in the body's circulation, leading to a decrease in the expression of tight junction (TJ) proteins such as zonula occludens (ZO)-1, occludin, and claudin as they interact with the barrier [25, 26]. Furthermore, hazardous chemicals can enter the intestine due to malfunctions of the intestinal barrier, impairing the immune system and promoting inflammation in the intestine, which can also lead to inflammatory bowel disease (IBD) [14]. Several studies have highlighted that an HFD has a negative impact on human health based on its association with obesity, type 2 diabetes, hypertension, IBD, leaky gut, and cardiovascular diseases [25, 27-29]. Previous studies have shown that consistent dietary fat consumption can lead to metabolic disorders, including insulin resistance and elevated levels of triglycerides (TGs), total cholesterol (TC), low-density lipoprotein cholesterol, high-density lipoprotein cholesterol, and serum glucose [30]. In rodent studies, feeding with a 60% fat diet may induce inflammation in adipose tissues, which is a significant component of metabolic syndrome [31]. Moreover, the microbiota composition increases the *Firmicutes*/*Bacteroides* ratio of the intestinal microbial group, and the abundance of beneficial bacteria in the intestines, such as *Lactobacillus* and *Akkermansia*, tends to decrease [25]. Numerous research studies have documented alterations in the intestinal microbiota of mice subjected to a HFD. The majority of these investigations have demonstrated a notable elevation in the abundance of *Firmicutes*, coupled with a tendency towards a reduction in *Bacteroides*. Additionally, an augmentation in the presence of *Oscillibacter* and *Desulfovibrionaceae*, both of which are Gram-negative bacteria known for endotoxin production, was observed [32, 33]. Conversely, a decline in the levels of *Cytophaga* and *Akkermansia*, members of the *Bacteriodetes* and *Verrumicrobia* phylum, was also noted [34].

Table 1 presents various HFD-induced dietary interventions in mouse studies regarding the development of metabolic disorders, alterations in gut microbiota, changes in intestinal permeability, and modifications in TJ proteins.

### High-Carbohydrate Diet (HCD)

Carbohydrates can be categorized into two groups: digestible and non-digestible carbohydrates. Digestible carbohydrates encompass starch and various sugars, such as fructose, glucose, lactose, and sucrose [47]. Carbohydrates are one of the primary sources of energy for humans. However, consuming excessive amounts of refined carbohydrates and added sugar can disrupt the balance of the intestinal microbiota, leading to gut dysbiosis. These types of carbohydrates are easily digestible and can promote the growth of beneficial bacteria such as *Lactobacillus*, *Bifidobacterium*, and *Akkermansia*. Recent research has shown that consuming highly refined carbohydrates can increase the production of proinflammatory cytokines and decrease the gene expression of TJ proteins [48-50]. Also, multiple research studies have demonstrated that the gut microbiota of mice fed a HCD undergoes changes, including an increase in the *Proteobacteria* phylum [51]. The presence of *Desulfovibrionaceae* was notably higher in the cecal content of the glucose group, suggesting that consuming glucose leads to a shift in microbiota composition, leading to a pro-inflammatory profile [52]. Consuming mono-and disaccharides such as glucose, fructose, and sucrose can result in dyslipidemia and comparatively higher blood glucose levels, endotoxin levels, fat mass, and glucose intolerance [49]. A study showed that when C57BL/6J mice were fed with 30% of fructose for 16 weeks, non-alcoholic fatty liver and liver inflammation were induced [53]. These conditions also accompany various chronic diseases such as obesity, type 2 diabetes, IBD, cardiovascular diseases, and dysbiosis [49, 50].

Artificial sweeteners, such as saccharin, aspartame, and sucralose, commonly utilized as sugar substitutes in food and beverages, are popular due to their low-calorie effects. Previous studies have indicated that non-caloric sweeteners can alter the composition of gut microbiota, potentially leading to disruptions in metabolic health. For instance, in a mouse study, the administration of aspartame at a dosage of 5–7 mg/kg/day was associated with increased fasting blood glucose levels and insulin resistance, which can contribute to chronic health conditions [54]. These changes in gut microbiota involve a reduction in beneficial bacteria, such as *Lachnospiraceae* and *Ruminococcaceae*, along with an increase in harmful bacteria, potentially resulting in microbial community disruption, IBD, and metabolic disorders, such as type 2 diabetes [55]. In prior research, the consumption of neotame for 4 weeks led to an increase in *Bacteroides* and other unspecified genera in male CD-1 mice, particularly within the *Bacteroides* phylum. Certain components of the *Lachnospira* and *Luminococcus* families in neotame-treated animals exhibited a notable decrease in *Blautia*, *Dorea*, *Oscillospira*, and *Luminococcus* compared to the control group [56]. Furthermore, the six-week administration of sucralose resulted in an increase in *Firmicures*, *Clostridium symbiosum*, and *Peptostreptococcus anaerobius* [57]. These findings indicate that artificial sweeteners disrupt the composition and diversity of the gut microbiome. Table 2 presents various HCD-induced dietary interventions in mouse studies regarding the development of metabolic disorders, alterations in gut microbiota, changes in intestinal permeability, and modifications in TJ proteins. Nevertheless, a more comprehensive understanding of the relationship between artificial sweeteners and gut microbiota necessitates further research.

## Leaky Gut-Linked Metabolic Diseases

A leaky gut commonly emerges as a consequence of impaired intestinal barrier function in chronic conditions [65]. It is a classic indicator of intestinal inflammation, and when the intestinal barrier is compromised, toxins can enter the bloodstream. These toxins can trigger inflammatory responses that may manifest as various diseases. A leaky gut could either be an underlying factor contributing to a disease or a result of the disease itself, such as in the case of liver disease. Both normal and imbalanced microbiota can play a role in causing inflammation or other outcomes that affect the disease [66].

## Obesity

Obesity is characterized by a body mass index (BMI) exceeding 30 kg/m^2^, which arises from the excessive accumulation of adipose tissues [67]. Obesity is one of the global severe social problems [68]. It is linked to several risk factors for gastrointestinal diseases, indicating potential impairments in gut health [69]. Recently, there has been a growing interest in the relationship between gut microbiota composition and obesity. Adipose tissues play a crucial role in regulating energy balance and lipid metabolism through the secretion of various peptide hormones, such as adiponectin, leptin, resistin, and tumor necrosis factor-α (TNF-α)[70]. Many disorders, including but not limited to type 2 diabetes, non-alcoholic steatohepatitis (NASH), dyslipidemia, and various other illnesses, are caused by obesity. As microorganisms can obtain energy from indigestible dietary components, the gut microbiota plays a crucial role in the development of obesity [71]. Gut microbiota is recognized as a source of increased plasma endotoxins associated with obesity and insulin resistance through elevated intestinal permeability in animal models [72], primarily including LPSs with a high affinity for lipoproteins [73]. Both in humans and animal models, obesity is associated with factors such as reduced bowel motility [74], nutritional deficiencies [75, 76], bacterial overgrowth [77], and changes in microbiota composition leading to the increased production of short-chain fatty acids (SCFAs) [78, 79], compromised barrier function with heightened bacterial translocation [80], and the development of gastrointestinal conditions, such as gastroesophageal reflux disease [81] and gallstone formation [82]. Moreover, obesity has been shown to affect inhibitory and excitatory enteric neurons [83]. These various factors collectively contribute to a potential deterioration in gut barrier function.

## Nonalcoholic Fatty Liver Disease (NAFLD)

Internationally, NAFLD is the most prevalent chronic liver disease [84]. The mechanisms underlying its development and progression are influenced by genetic factors and metabolic dysregulation, including type 2 diabetes and insulin resistance, and are closely related to high energy intake [85]. A Western-style diet, which is rich in cholesterol, can lead to the development of fatty liver through exposure to inflammatory cytokines, insulin resistance, and oxidative stress [86]. The accumulation of TGs and fats in the liver leads to the development of NAFLD, which can advance from simple steatosis to NASH, cirrhosis, and hepatocellular cancer [87].

NAFLD can be caused by weight gain, obesity, and lack of physical activity [88]. The hepatic portal vein is anatomically and physiologically connected to the intestine [89]. Recently, there has been a growing focus on the dysfunction of the gut-liver axis, which encompasses dysbiosis, bacterial overgrowth, and alterations in intestinal permeability, all of which are linked to the advancement of NAFLD [90]. For example, alterations in both the quantity and composition of bacteria disrupt the intestinal barrier, enabling bacterial translocation and subsequently triggering an inflammatory reaction [91]. The gut-liver axis refers to the symbiotic link between the gut, its microbiota, and the liver. This connection develops as a result of interactions between signals produced by nutritional, genetic, and environmental factors [92]. When the function of this axis is compromised, it leads to the translocation of bacteria and microbial components, along with metabolic by-products, to the liver due to the imbalance in gut microbiota composition and changes in mucosal permeability [27]. The changes in the gut microbiota linked to NAFLD are closely related to the clinical stage of the disease [93].

In a previous study, a cohort of 16 individuals diagnosed with NASH and a control group of 22 healthy individuals provided fecal samples for analysis [94]. The results of the investigation revealed a significant reduction in the prevalence of *Faecalibacterium* and *Anaerosporobacter* in the fecal samples of individuals with NASH. Conversely, there was an elevated abundance of *Parabacteroides* and *Allisonella* in the same group of individuals. Recent studies have shown that the proportion of *Akkermansia muciniphila*, a member of the *Verrucomicrobia* phylum, is reduced in individuals with obesity and diabetes, as well as in corresponding mouse models. Additionally, it has been found that the presence of *A. muciniphila*, regardless of its viability, is linked to improvements in the integrity of the intestinal mucosal barrier, an increase in goblet cells, and enhanced metabolic functions [95]. The gut-liver axis plays a pivotal role in the development of NAFLD, primarily by mediating communication between the intestinal microbiota and the host's immune system, which regulates inflammation, insulin resistance, and intestinal permeability. Still, comprehensive randomized trials utilizing antibiotics, probiotics, and prebiotics, along with imaging and histological assessments, are imperative [96].

## Brain Dysfunction

The interaction between the brain and the gastrointestinal tract has been widely acknowledged in scientific literature, as it involves various physiological systems, such as the autonomic neural network, the enteric neural network, the neuroendocrine neural network, and the immune network [97]. In recent studies, researchers have discovered a new link between the gut and the liver, which is influenced by the autonomic nervous system and is triggered by the gut microbiota [98]. The intestinal microbiota is a crucial component of the gut-brain neuroendocrine metabolic axis, and the use of antibiotics can disrupt its stability, potentially leading to inflammation [99]. Inflammation is a key factor related to the blood-brain barrier (BBB) in the brain. To understand the relationship between gut and brain leakiness, it is essential to comprehend the neurovascular barrier under normal physiological conditions, which plays a role in limiting BBB permeability and preventing the entry of substances like bacteria into the brain. However, inflammation and physiological stressors disrupt the BBB, impairing its selective substance passage. A leaky gut may be one of the fundamental causes associated with the breakdown of the BBB, and conditions like hypoxia and inflammation are known to increase the intercellular permeability of the BBB [100].

There are various pathways through which the gut microbiome and the brain communicate, and these pathways can be affected by changes in the levels of certain substances such as SCFAs, tryptophan (which is a precursor of dopamine and serotonin), gamma-aminobutyric acid, and other amino acids [101]. The metabolic by-products of the gut microbiota have garnered significant attention, especially SCFAs, which are produced by anaerobic microbes in the caecum, such as Enterococcus. These SCFAs are generated through the fermentation of dietary fiber, which consists of non-digestible carbohydrates. The presence of SCFAs, as well as the levels of colonic Enterococcus, have been linked to various beneficial effects on the host, including the suppression of appetite [102]. Additionally, SCFAs can traverse the intestinal barrier, enter the systemic circulation, cross the BBB, and reach the brain parenchyma [103]. This allows them to directly influence the hypothalamic regulation of metabolism and appetite [104]. The production of SCFAs by the gut microbiota has been found to have an impact on the maintenance of the BBB by promoting the synthesis of TJ proteins [105]. This enhanced integrity of the BBB serves to restrict the passage of unwanted metabolites into the brain tissues [106]. The lipoproteins and LPSs produced by the harmful gut microbiota can impact autoimmune function by inducing the release of cytokines from immune cells. These cytokines can traverse the BBB and activate neurons, leading to modifications in neurological functions and consequently affecting mood and behavior [107]. The gastrointestinal system can indeed impact the BBB by releasing gastrointestinal-derived hormones, facilitating the passage of certain drugs, amino acids, and small molecules across the barrier. This interaction can also affect cytokine production, a crucial aspect of innate immune system activation [108]. While various studies have provided evidence of the potential involvement of leaky gut in the onset of diseases, further exploration is needed to elucidate the precise physiological effects and mechanisms.

## Conclusion

This literature review presents current research findings on the relationship between obesity and metabolic disorders caused by HFD and HCD, as well as disruptions in the gut microbiota. Additionally, we discussed the effects of these factors on intestinal microorganisms and the communication pathways between the gut, liver, and brain. Consuming a diet rich in fats and carbohydrates can weaken the adherence of TJ proteins in the gastrointestinal tract. Consequently, this allows endotoxins, such as LPSs produced by detrimental bacteria, to permeate through portal veins. This process can result in metabolic endotoxemia, which disrupts the composition of the gut microbiome and diminishes the production of metabolites like SCFAs. However, the precise correlation between gut microbiota and alternative sweeteners remains uncertain, thus necessitating additional investigation in this domain. The investigation of the impact of diet on gut microbiota and the underlying mechanisms of the gut-liver and gut-brain axis is of significant importance. However, the limited amount of research conducted on the gut-liver axis poses challenges in fully understanding the intricate connection between diet and the gut-brain axis. Hence, there is a pressing need for comprehensive research endeavors aimed at elucidating the intricate gut-brain mechanisms that underlie intestinal health and microbiota.

## Figures and Tables

**Table 1:** Metabolic disorders, alterations in gut microbiota and permeability, and changes in tight junction (TJ) proteins confirmed through high-fat diet (HFD)-induced dietary interventions in mouse studies.

Animal	Diet composition	Duration	Disorder	Disease	Microbiota	Gut permeability	TJ protein/mucin production	Reference
C57BL/6J mice, male	60% HFD	HFD for 5 weeks; low-fat diet for 2 weeks	Fasting blood glucose, hepatic lipid accumulation, hepatic TG ↑	_	*Firmicutes*/*Bacteroidetes* (F/B) ratio ↑	↑	_	[35]
C57BL/6 mice, male	60% HFD	10 weeks	HDL ↓, gut barrier dysfunction, hyperlipemia, TG/TC/LDL ↑	Obesity	*Clostridium sensu stricto* 1 ↑	_	_	[28]
C57BL/6, male	60% HFD	4 weeks	Insulin sensitivity, glucose tolerance ↑	Obesity	*Bacteroidal Clostridiales* spp ↑ *Bacteroidal* ↓	_	_	[36]
C57BL/6J mice, male	60% HFD	8 weeks	Insulin resistance, hyperglycemia ↑	Obesity, type 2 diabetes	*Akkermansia* ↓	↑	Goblet cells number↓	[37]
C57BL/6J mice, male	60% HFD	4 weeks	Glucose intolerance ↑	Metabolic disease, obesity, type 2 diabetes	Clostridia class, *Bacteroidales* S24-7 family, and *Candidatus arthromitus* ↓ *Erysipelotrichi* class, *Desulfovibrionales* ↑	↑	Claudin-7↓	[38]
C57BL/6J mice, male	60% HFD	20 weeks	Glucose intolerance, insulin resistance, adipose tissue inflammation ↑	_	*Bilophila wadsworthia* spp. ↑	↑	Claudin-1, occludin ↓	[39]
C57BL/6J mice, female	60% HFD	8 or 12 weeks	HOMA-IR, insulin resistance ↑	_	*Bacteroidetes*: *Firmicutes* ↑	↑	ZO-1 ↓	[32]
C57BL/6J mice, male	60% HFD	120 days	Insulin resistance, hyperglycemia, hepatic lipid accumulation, TG/TC ↑	Obesity	*Bifidobacterium genus* ↓, *Enterobacteriaceae* family ↑	↑	Micelles per enterocyte ↑	[40]
Swiss albino mice, male	60% HFD	8 weeks	Insulin resistance, hepatic lipid accumulation ↑	_	_	↑	ZO-1, claudin-2, occludin ↓	[26]
SD rats, male	45% HFD with 1.25% cholesterol	8 weeks	TG/TC/LDL/AST/ALT ↑	Hepatic steatosis, hyperlipidemia	*Firmicutes*, *Desulfovibrionaceae* ↑	↑	ZO-1, Muc2, occludin ↓	[33]
Swiss mice, male	45% Unmodified fat	8 weeks	Glucose homeostasis ↑	Cardiovascular disease, dyslipidemia, atherogenesis	_	↑	Goblet cells, Muc2 ↓	[41]
C57BL/6 J mice, male	60% HFD	5 weeks	Insulin resistance, basal glycemia ↑	Hypertension	*Proteobacteria*, F/B ratio ↑, *Akkermansia* ↓	↑	ZO-1, Muc2, occludin, Muc3 ↓	[34]
C57BL/6J mice, male	60% HFD	12 weeks	Serum glucose ↑	Type 2 diabetes, hypertension, cardiovascular diseases	F/B ratio ↑	_	_	[42]
C57BL/6 mice, male	HFD (45% kcal/fat (39% lard+6% soybean oil)	16 weeks	Bone loss ↑	Leaky gut, osteoporosis, obesity	*Lactobacillus* ↓, F/B ratio ↑	↑	Claudin-1, claudin-15, ZO-1, JAM-A ↓	[43]
C57BL/6J male mice, male	60% HFD	16 weeks	Insulin resistance, blood glucose ↑	Hyperlipidemia	*Faecalibaculum*, F/B ratio ↑, *Parasutterella* ↓	↑	ZO-1, occludin, claudin-1 ↓	[25]
C57BL6/J mice, male	HFD (lard (20 g % diet)	60 days	Insulin resistance, hyperinsulinemia ↑	Type 2 diabetes, obesity	_	↑	ZO-1, claudins-1, -2, -3 ↓	[44]
C57BL/6 mice	60% HFD	6 weeks	Insulin resistance ↑	IBD, leaky gut	*Firmicutes*, *Actinobacteria* ↑	↑	Occludin, goblet cell ↓	[29]
SD rats, male	60% HFD	8 weeks	Insulin resistance, TG ↑	NAFLD	*Lactobacillaceae*, *Lachnospiraceae* ↓	↑	Claudin-1 ↓	[45]
C57BL/6 mice, male	60% HFD	12 weeks	Insulin resistance, metabolic endotoxemia ↑	Obesity, diabetes	*Firmicutes* ↑	↑	Occludin, ZO-1, Claudin-5↓	[46]

TG, triglycerides; HDL, high-density lipoprotein cholesterol; TC, total cholesterol; LDL, low-density lipoprotein cholesterol; AST, aspartate aminotransferase; ALT, Alanine aminotransferase; F/B, *Firmicutes*/*Bacteroidetes*; ZO-1, zonulin-1; JAM-A, junctional adhesion molecule; Muc2, mucin 2; Muc3, Mucin 3.

**Table 2:** Metabolic disorders, alterations in gut microbiota and permeability, and changes in tight junction (TJ) proteins confirmed through high-carbohydrate diet(HCD)-induced dietary interventions in mouse studies.

Animal	Diet composition	Duration	Disorder	Disease	Microbiota	Permeability	TJ protein/mucin production	Reference
C57BL/6J mice, female	65% fructose	12 weeks	Endotoxin translocation, intestinal barrier impairment ↑	ￚ	*Enterobacteriaceae*, *Coprococcus*, *Ruminococcus*↑/ *Bacteroidetes*↓	↑	Occludin, claudin-2, claudin-5 ↓	[48]
C57BL/6J mice, male	30% fructose		Glucose homeostasis, insulin sensitivity, hepatic lipid accumulation, TG ↑	Hepatic steatosis	*Desulfovibriovulgaris*, *Proteobacteria*↑	↑	ZO-1, occludin ↓	[50]
C57BL/6J mice	30% fructose	8 weeks	Insulin resistance, blood glucose, TG ↑	Hepatic steatosis	ￚ	↑	Occludin, ZO-1 ↓	[58]
C57BL/6N mice, male	30% fructose	8 weeks	Insulin resistance, hippocampal neuroinflammation, intestinal epithelial barrier damage ↑	ￚ	*Bacteroidetes*↓/ *Proteobacteria*, *Deferribacteraceae*, *Helicobacteraceae*↑	↑	ZO-1 ↓	[59]
Fischer 344 rats, female	30% fructose	8 weeks	Insulin resistance, TG ↑	NAFLD, NASH, fibrosis, leaky gut	*Bacteroidetes*, *Proteobacteria*, *Escherichia*↑/*Lactobacillus*, *Akkermansia*↓	↑	ZO-1, occludin, claudin-1, claudin-4 ↓	[60]
Fisher 344 rats, male	20% fructose	10 weeks	Insulin resistance, hepatic fat ↑	Liver fibrosis, hepatocarcinogenesis	ￚ	↑	ZO-1 ↓	[61]
C57BL/6 mice, male	15% fructose	9 weeks	Glucose homeostasis, insulin sensitivity, TG ↑	Liver steatosis, liver damage	*Firmicutes* ↑	ￚ	Occludin, ZO-1, claudin-2 ↓	[52]
C57BL/6J mice, female	30% fructose	16 weeks	Insulin resistance, cholesterol ↑	NAFLD	ￚ	↑	ZO-1, occludin ↓	[53]
C57BL/6J mice, male	10% fructose	10 weeks	Insulin resistance, TG, TC, LDL-C ↑	Obesity, hyperphagia	*Proteobacteria*, *Bacteroides*↑ / *Lactobacillus*, *Bifidobacterium*↓	ￚ	ￚ	[51]
C57BL/6 mice, male	15% glucose	9 weeks	Glucose intolerance, hyperglycemia ↑	-	*Proteobacteria*, *Desulfovibrionaceae*, *Desulfovibrionaceae*↑	ￚ	ￚ	[52]
C57BL/7 mice, male	65% glucose	12 weeks	Glucose homeostasis, insulin sensitivity, TG ↑	Hepatic steatosis, inflammation	*Enterobacteriaceae*, *Coprococcus*, *Ruminococcus*↑/ *Bacteroidetes*↓	↑	Occludin, ZO-1 ↓	[50]
Wild-type mice 129S1/SvimJ, female	50% sucrose	10 days	SCFAs ↓	IBD	*Verrucomicrobia*↑ / *Lachnospiraceae*, *Prevotellaceae*, *Anaeroplasmataceae*↓	↑	ￚ	[62]
CD-1 mice, male, female	Acesulfame-potassium (37.5 mg/kg/day)	4 weeks	Glucose intolerance, chronic inflammation, energy homeostasis ↑	Type 2 diabetes mellitus	Males: ↑ *Bacteroides*, *Anaerostipes*, *Sutterella* Females: ↑ in fecal *Mucispirillum*; ↓ *Lactobacillus*, *Clostridium*, an unassigned *Ruminococcaceae* genus	ￚ	ￚ	[63]
SD rats, male	Aspartame (5–7 mg/kg/day)	8 weeks	Insulin tolerance, systemic inflammation, fasting glucose ↑	Type 2 diabetes	Normal rats: ↑ fecal *C. leptum* Obese rats: ↑ fecal total bacteria, *Bifidobacterium* spp., *Enterobacteriaceae*, *C. leptum*, and *Roseburia* spp	ￚ	ￚ	[54]
C57BL/7 mice, male	Saccharin (0.1 mg/kg/day)	11 weeks	Glucose intolerance ↑	-	↑ fecal abundance *Bacteroides*, *Clostridiales* ↓ fecal *L. reuteri*, members of *Clostridiales*	ￚ	ￚ	[64]
CD-1 mice, male	Neotame 0.75 mg/kg/day	4 weeks	Cholesterol, campesterol, stigmastanol ↑	IBD, type 2 diabetes	*Bacteroides* and undefined genus in family S24-7↑, *Lachnospiraceae* and *Ruminococcaceae* families (*e.g.*, *Blautia*, *Dorea*, *Oscillospira*, and *Ruminococus*) ↓	ￚ	ￚ	[56]
C57BL/6 mice, male	Sucralose 0.0003 mg/mL	16 weeks	Blood glucose ↑	IBD, NAFLD, IBS	*Lachnoclostridium*, *Lachnospiraceae*↓/ *Allobaculum*, *Tenacibaculum*, *Ruegeria*, *Staphylococcus*↑	ￚ	Muc2 ↓	[55]
C57BL/6 mice	Sucralose 1.5 mg/ml	6 weeks	Gut damage ↑	IBD	*Ficmicures*, *Actinomycetes*, *Peptostreptococcus stomatis*, *Clostridium*, *symbiosum*, *Peptostreptococcus* ↑ / *proteobacteria* ↓	↑	Occludin, claudin-1, claudin-4 ↓	[57]

TG, triglycerides; TC, total cholesterol; LDL, low-density lipoprotein cholesterol; SCFAs, short-chain fatty acids; NAFLD, nonalcoholic fatty liver disease; NASH, nonalcoholic steatohepatitis; pIBD, inflammatory bowel disease; IBS, inflammatory bowel syndrome; ZO-1, zonulin-1; Muc2, mucin 2.
